# Effects of phased focused nursing on liver function and self-care ability in chronic hepatitis B patients

**DOI:** 10.3389/fmed.2025.1679180

**Published:** 2026-01-06

**Authors:** Dongcang Hou, Fei Liu, Dongjie Hou, Chunrong Ping, Wenying He, Zeyang Chen, Ganlu Tian, Tian Qin, Wenjing Zhou, Menghui Xu, Xiaoman Ruan

**Affiliations:** 1 Department of Gastroenterology, Second Hospital of Hebei Medical University, Shijiazhuang, Hebei, China; 2 Department of Rheumatology and Immunology, Hebei Provincial Hospital of Traditional Chinese Medicine, Shijiazhuang, Hebei, China

**Keywords:** chronic hepatitis B, phased focused nursing, liver function, self-care ability, treatment compliance

## Abstract

**Aim:**

Chronic hepatitis B (CHB) patients often face challenges related to liver function recovery, psychological wellbeing, and self-care ability during treatment. Conventional routine nursing may not comprehensively address these multi-dimensional issues. This study aimed to investigate the effects of phased focused nursing on liver function and self-care ability in patients with CHB.

**Methods:**

A total of 120 CHB patients who received treatment in our hospital from March 2023 to March 2024 were selected and randomly divided into a control group and a study group. The control group received routine nursing, while the study group received phased focused nursing in addition to routine nursing. Liver function, psychological resilience, self-care ability, subjective wellbeing, quality of life, sleep quality, treatment compliance, and nursing satisfaction were compared between the two groups before and after the intervention.

**Results:**

After the one-month intervention, the levels of aspartate aminotransferase (AST), alanine aminotransferase (ALT), and total bilirubin (TBil) were lower in the study group than in the control group (*p* < 0.01). In addition, the scores of the Connor–Davidson Resilience Scale (CD-RISC), Exercise of Self-Care Agency (ESCA) scale, and World Health Organization Quality of Life-BREF (WHOQOL-BREF) scale were higher in the study group than in the control group (*p* < 0.01). Compared to the control group, the study group had higher scores for positive emotions and lower scores for negative emotions on the Memorial University of Newfoundland Scale of Happiness (MUNSH) after the one-month intervention (*p* < 0.01). Furthermore, the Pittsburgh Sleep Quality Index (PSQI) scores in the study group were lower compared to the control group (*p* < 0.01). Compared to the control group, treatment compliance and nursing satisfaction were higher in the study group (*p* < 0.01 and *p* = 0.01).

**Conclusion:**

Phased focused nursing can significantly improve liver function and has a profound impact on enhancing psychological resilience, sleep quality, self-care ability, subjective wellbeing, and quality of life. Additionally, it enhances treatment compliance and increases nursing satisfaction, indicating its superiority over routine nursing in the multidimensional management of CHB patients.

## Introduction

Chronic hepatitis B (CHB) is a prevalent chronic infectious disease encountered in clinical practice (1). As the disease progresses, it can gradually advance to liver cirrhosis, liver cancer, liver failure, and other life-threatening conditions, severely endangering the patient’s life (2). According to the World Health Organization, there are 240 million CHB cases worldwide. In China, there are approximately 90 million hepatitis B virus carriers, of whom 28 million have CHB, accounting for one-third of the global total (3). Currently, there is no definite and effective treatment for CHB in clinical practice; it can only be managed through long-term medication (4). Studies have shown that improving patients’ self-care ability and enhancing their confidence in treatment can effectively improve the efficacy of drug therapy (5). However, due to the prolonged course and severity of the disease, patients often have a limited understanding of CHB, resulting in low treatment compliance and poor self-care ability. Moreover, routine nursing interventions for CHB patients fail to stimulate patients’ enthusiasm and pay insufficient attention to their psychological state, which, in turn, affects the treatment outcome (6). Therefore, there is an urgent need for more effective intervention measures to improve the prognosis of CHB patients.

Focused nursing intervention emphasizes psychological support and aims to explore and awaken the potential of patients to enhance their self-care ability (7). In recent years, phased focused nursing intervention has been widely implemented in chronic disease management; however, its use in CHB patients has rarely been reported (8, 9). Therefore, this study aimed to assess the effects of phased focused nursing on liver function and self-care ability in CHB patients.

## Data and methods

### Study design

A total of 120 CHB patients who received treatment in our hospital from March 2023 to March 2024 were selected and randomly divided into a control group and a study group, with 60 patients in each group. The inclusion criteria were as follows: (1) Patients who met the diagnostic criteria outlined in the 2019 “Guidelines for the Prevention and Treatment of Chronic Hepatitis B,” with hepatitis B surface antigen (HBsAg) and/or hepatitis B virus DNA positivity for more than 6 months; (2) no manifestations of portal hypertension or other signs of liver cirrhosis; and (3) age ≥18 years. The exclusion criteria were as follows: (1) Patients with heart, lung, or renal failure; (2) patients with systemic malignant tumors; (3) patients with mental cognitive disorders; and (4) patients with autoimmune liver disease. All patients provided informed consent, and this study was approved by the hospital’s Medical Ethics Committee.

### Methods

The control group received routine nursing, including psychological support, lifestyle guidance, dietary management, and complication prevention.

In addition to routine nursing, the study group received phased focused nursing. The specific components were as follows:

1. Establishment of a focused nursing intervention group. A total of five members were selected, including one supervisor, two nurses, one psychological consultant, and one attending doctor. The supervisor served as the group leader, whose primary responsibilities were planning, coordination, and supervision. The two nurses were responsible for implementing focused nursing interventions and collecting data, the attending doctor was responsible for formulating the clinical plan for patients, and the psychological consultant was responsible for providing psychological care to patients. Before the intervention, the team members received professional training and were evaluated.
2. Implementation of phased focused nursing. The entire intervention was conducted over a 1-month period. Patients were hospitalized for the initial 3-day assessment and intervention phase, followed by follow-up care at home for the remaining 27 days.

Stage 1: Assistance, assessment, and problem identification. On the day of admission, the nurse engaged in a one-on-one conversation with each patient, which lasted approximately 30 min. During this conversation, the nurse performed an admission assessment to gain a detailed understanding of the patient’s knowledge and perceptions of CHB and made detailed records of the findings. The nurse appropriately guided the patient to recall past conditions and worked to alleviate any negative emotions associated with past experiences. This was achieved through gentle verbal encouragement and reassurance. The nurse acknowledged the patient’s existing disease knowledge and self-care ability, promptly corrected any misconceptions about the disease, and guided the patient in acquiring an accurate understanding of CHB and proper self-care methods. The nurse also assessed the patient’s psychological state, engaged in discussions about various methods with the patient through communication and guidance, acknowledged the patient’s suggestions, and enhanced the patient’s confidence in self-care.

Stage 2: Target-setting stage for helping patients set achievable goals. This stage occurred on the second day of admission and lasted approximately 45 min. Considering the patient’s disease characteristics and psychological traits, the nurse identified and assisted the patient in setting feasible goals. The guiding principle was to set smaller, short-term goals rather than larger ones to enhance the patients’ self-confidence. After setting feasible goals, the nurse provided several alternative options to the patients. Once a particular option was selected, it remained changed. To further clarify the goals, the nurse could use visual aids, such as simple charts or diagrams, to explain the target values. After the three-day in-hospital assessment and initial goal-setting, patients were discharged and continued the subsequent stages at home.

Stage 3: Exploration of exceptions. This stage was conducted on the third day of admission and lasted approximately 60 min. Psychological suggestion methods were used to help patients recall the past factors that contributed to their high psychological resilience, such as family support and social support. The nurse could ask open-ended questions such as “Can you think of a time when you felt really supported by your family during a difficult time?” to stimulate the patient’s memory. In addition, patients were encouraged to reflect on their self-care patterns and capabilities during previous periods of occasional symptom relief. They were required to think independently and fill out questionnaires, which usually took approximately 20 min. Moreover, the nurses and patients jointly discussed successful coping cases. The nurse inquired about which health strategies had helped the patients maintain these benefits, clarified the criteria for achieving the goals, helped the patients in establishing determination to achieve the goals, and assisted them in identifying the impact of adopting positive coping strategies on the management of CHB.

Stage 4: Feedback stage. This stage occurred once a week during the 27-day home-based intervention period, and each feedback session lasted approximately 30 min. During the implementation of the intervention measures, the nurses and patients jointly analyzed the achievement of the target values for self-care ability and psychological resilience. The nurses acknowledged the efforts of the patients and helped them make progress, encouraging them to continue to strive toward the target values. The nurse could use positive reinforcement statements, such as “You’ve made great progress this week, keep it up!” To ensure the patients’ adherence at home, the nurses also made regular phone calls to monitor progress, answer questions, and provide additional support.

Stage 5: Evaluation stage. This stage took place at the end of the one-month intervention, lasting approximately 40 min. The nurses asked the patients whether they were satisfied with their scores for the target values this time, whether they could obtain higher scores, and whether they could achieve higher scores in the next evaluation. They also thanked the patients for their efforts and cooperation to stimulate their potential.

Both groups received interventions for 1 month. The study group underwent an initial three-day in-hospital phase followed by 27 days of home-based care, while the control group received routine nursing throughout the 1-month period, which could be either in-hospital or outpatient-based depending on the patient’s condition.

### Observation indicators

1. Liver function: Fasting peripheral venous blood was collected from the patients, and serum was obtained after centrifugation for 15 min. Levels of aspartate aminotransferase (AST), alanine aminotransferase (ALT), and total bilirubin (TBil) were measured using the Beckman Coulter AU5800 automatic biochemical instrument.
2. Psychological resilience: The Connor–Davidson Resilience Scale (CD-RISC) was used for evaluation (10). This scale included three items: tenacity, self-improvement, and optimism. The total score was 100 points. The higher the score, the better the psychological state of the patient, indicating higher psychological resilience.
3. Self-care ability: The Exercise of Self-Care Agency (ESCA) scale was used for evaluation (11). This scale included four items: health knowledge level, self-concept, self-responsibility, and self-care skills. The total score was 100 points. The higher the score, the greater the patient’s self-care ability.
4. Subjective wellbeing: The Memorial University of Newfoundland Scale of Happiness (MUNSH) was used for evaluation (12). The scale was divided into two dimensions: positive emotion and negative emotion, with score ranges of 0 ~ 26 and 0 ~ 22, respectively. The higher the score, the stronger the subjective wellbeing of the patient.
5. Quality of life: The World Health Organization Quality of Life-BREF (WHOQOL-BREF) scale was used for assessment (13). This scale included four dimensions: physiological, psychological, social relations, and environment. The higher the score, the better the patient’s quality of life.
6. Sleep quality: Pittsburgh Sleep Quality Index (PSQI) was used for evaluation (14). The total score ranged from 0 to 21. The higher the score, the worse the sleep quality.
7. The treatment compliance was evaluated using a self-developed scale, which categorized patients into three groups: complete compliance, where patients followed the medical staff’s requirements strictly during the intervention; partial compliance, where patients mostly followed the instructions but exhibited some irregular behaviors; and non-compliance, where patients refused to follow the medical staff’s instructions, refusing treatment and care. The total compliance rate was calculated by dividing the sum of the number of patients with complete and partial compliance by the total number of patients, then multiplying by 100%.
8. Nursing satisfaction: Patient satisfaction was evaluated using a self-developed questionnaire. The total score was 100 points, with scores ≥90 indicating “very satisfied,” scores ≥70 indicating “basically satisfied,” and scores <70 indicating “dissatisfied.” Overall satisfaction was calculated as follows: (number of very satisfied cases + number of basically satisfied cases)/total cases × 100%.

### Statistical analysis

SPSS 24.0 statistical software was used for data processing. We used the Shapiro–Wilk test to assess the normality of continuous variables. Measurement data confirmed to be normally distributed were expressed as (x ± s). When comparing the means of two independent groups, we used the independent samples *t*-test. For paired data, we used the paired samples *t*-test. Measurement data that did not follow a normal distribution were expressed as the median and interquartile range [M (Q1, Q3)]. When comparing two independent groups, we used the Mann–Whitney U test. For paired data that were not normally distributed, the Wilcoxon signed-rank test was used. Categorical data were expressed as percentages. To compare the proportions of categorical variables between the two groups, we used the χ^2^ test. A *p*-value of less than 0.05 was considered to indicate a statistically significant difference between the groups.

## Results

### Baseline characteristics of the patients in the two groups

There were no differences in the baseline characteristics of the patients between the two groups (*p* > 0.05, Table 1).

**Table 1:** Baseline characteristics of the patients in both groups.

Groups	Cases	Sex		Age (years)	Course of disease (years)
		Male	Female		
Control group	60	33 (55.00)	27 (45.00)	45.26 ± 4.68	3.74 ± 0.38
Study group	60	34 (56.67)	26 (43.33)	45.31 ± 4.72	3.77 ± 0.42
χ^2^/t		0.03		0.05	0.41
*P*		0.85		0.95	0.68

### Liver function between the two groups

**Figure 1:**
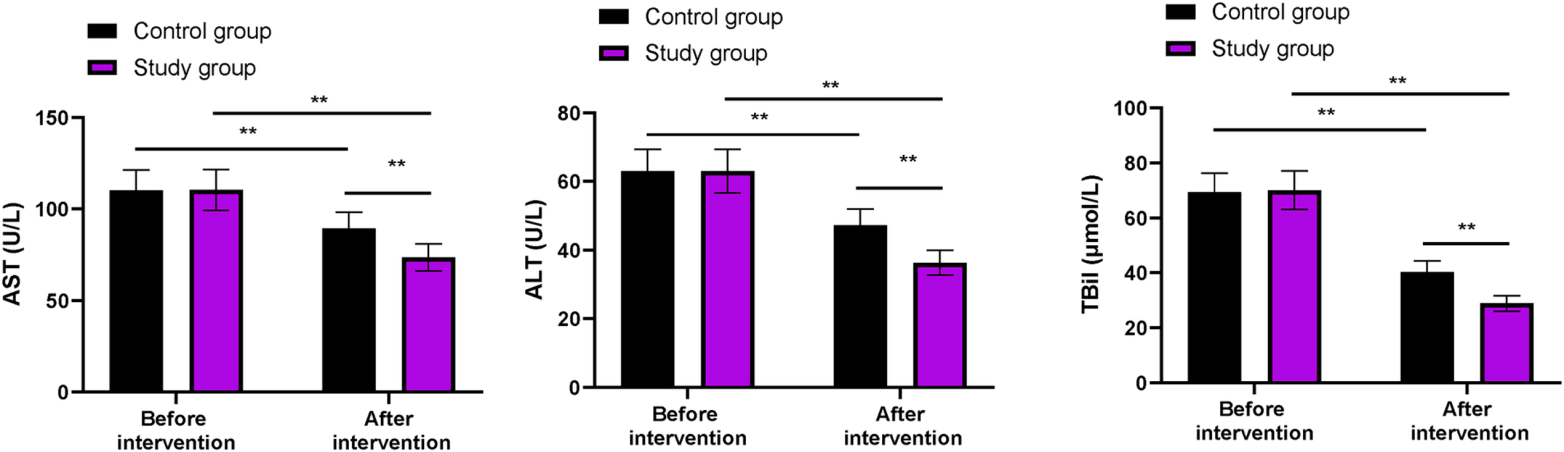
Liver function in both groups. ^**^p < 0.01; ns indicates that the difference was not significant.

Before the intervention, the levels of AST, ALT, and TBil showed no significant differences between the two groups (*p* > 0.05). After the intervention, the levels of AST, ALT, and TBil in both groups were lower than those before the intervention (*p* < 0.01). More importantly, compared to the control group, the levels of AST, ALT, and TBil were lower in the study group after the intervention (*p* < 0.01, Figure 1).

### Psychological resilience between the two groups

**Figure 2:**
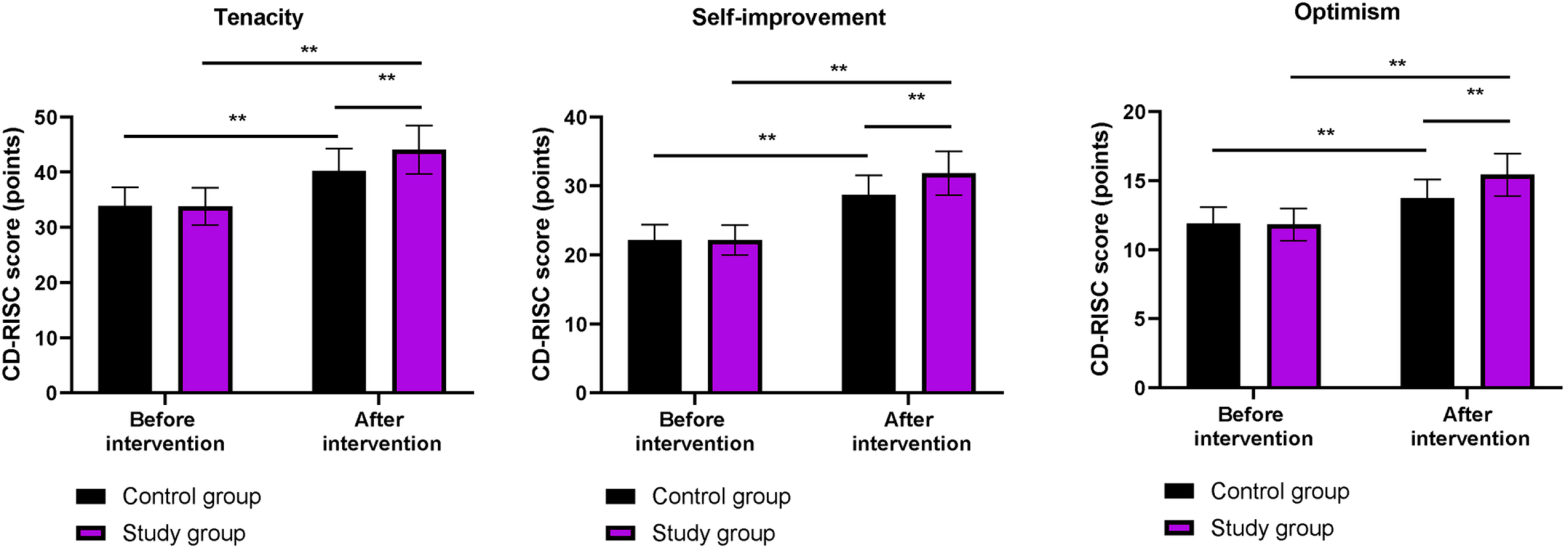
Psychological resilience in both groups. ^**^p < 0.01; ns indicates that the difference was not significant.

Before the intervention, the CD-RISC scores for tenacity, self-improvement, and optimism did not differ significantly between the two groups (*p* > 0.05). After the intervention, the CD-RISC scores in both groups were higher than those before the intervention (*p* < 0.01). More importantly, compared to the control group, the CD-RISC scores were higher in the study group after the intervention (*p* < 0.01, Figure 2).

### Self-care ability between the two groups

**Figure 3:**
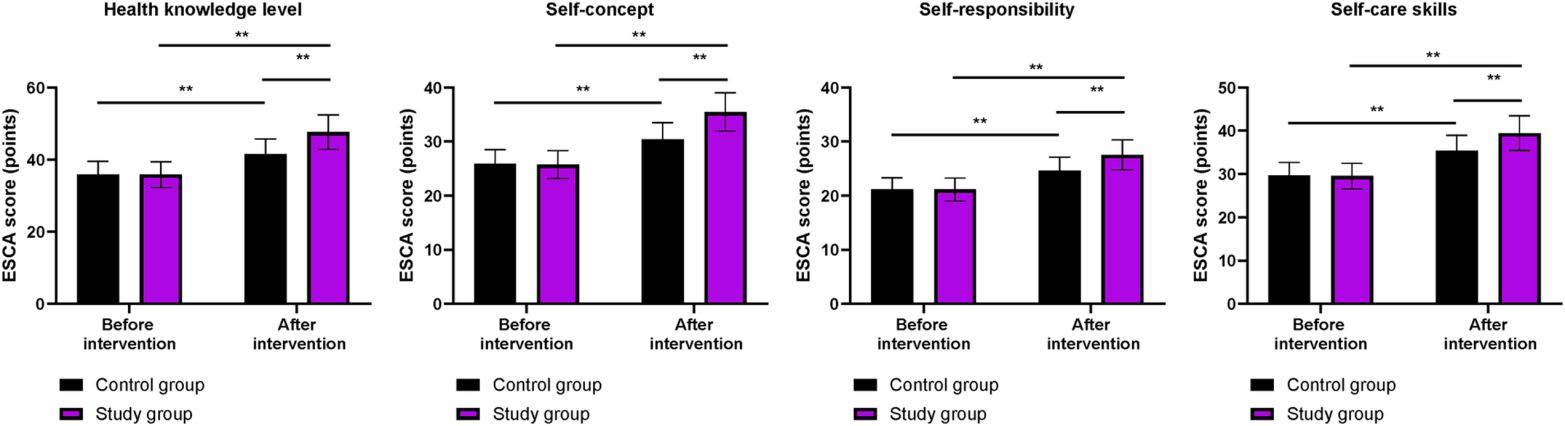
Self-care ability in both groups. ^**^p < 0.01; ns indicates that the difference was not significant.

Before the intervention, the ESCA scores for health knowledge level, self-concept, self-responsibility, and self-care skills did not differ significantly between the two groups (*p* > 0.05). After the intervention, the ESCA scores in both groups were higher than those before the intervention (*p* < 0.01). More importantly, compared to the control group, the ESCA scores were higher in the study group after the intervention (*p* < 0.01, Figure 3).

### Subjective wellbeing between the two groups

**Figure 4:**
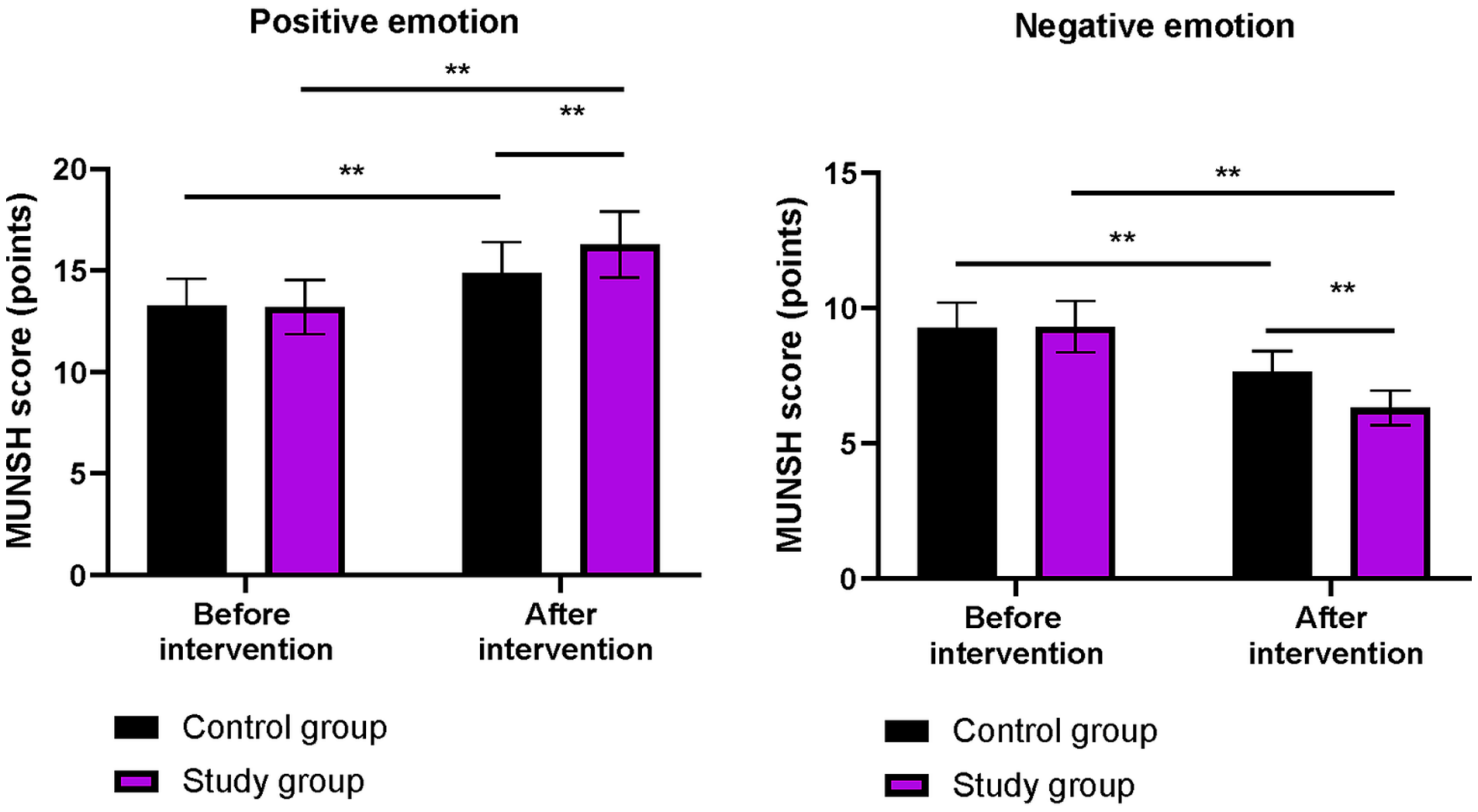
Subjective wellbeing in both groups. ^**^p < 0.01; ns indicates that the difference was not significant.

Before the intervention, the MUNSH scores for positive and negative emotions did not differ significantly between the two groups (*p* > 0.05). After the intervention, the scores of positive emotions on the MMUNSH in both groups were higher than those before the intervention, while the scores of negative emotions on the MUNSH in both groups were lower than those before the intervention (*p* < 0.01). More importantly, compared to the control group, the study group had higher positive emotion scores and lower negative emotion scores on the MUNSH after the intervention (*p* < 0.01, Figure 4).

### Quality of life between the two groups

**Figure 5:**
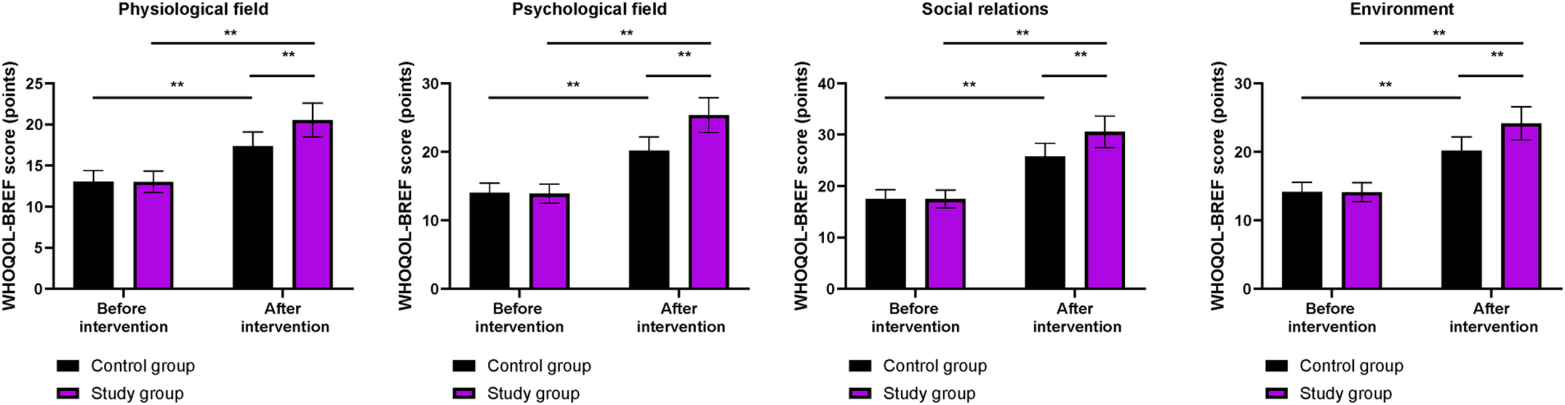
Quality of life in both groups. ^**^p < 0.01; ns indicates that the difference was not significant.

Before the intervention, the WHOQOL-BREF scores for physiological field, psychological field, social relations, and environment did not differ significantly between the two groups (*p* > 0.05). After the intervention, the WHOQOL-BREF scores in both groups were higher than those before the intervention (*p* < 0.01). More importantly, compared to the control group, the WHOQOL-BREF scores were higher in the study group after the intervention (*p* < 0.01, Figure 5).

### Sleep quality between the two groups

**Figure 6:**
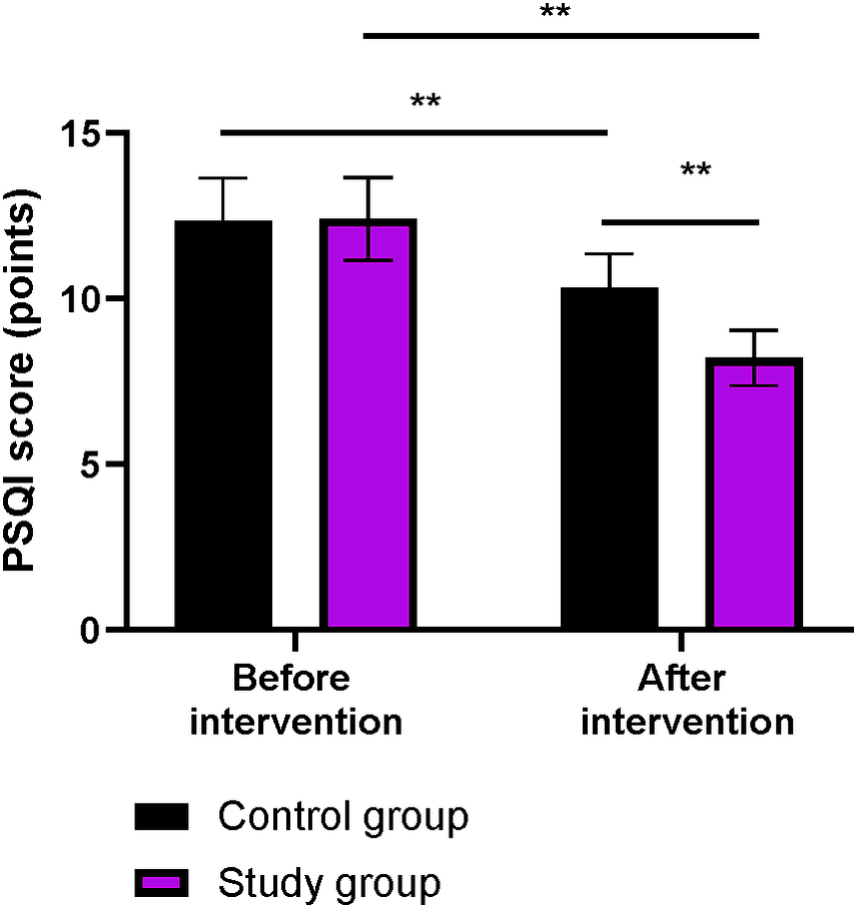
Sleep quality in both groups. ^**^p < 0.01; ns indicates that the difference was not significant.

Before the intervention, the PSQI score did not differ significantly between the two groups (*p* > 0.05). After the intervention, the PSQI scores in both groups were lower than those before the intervention (*p* < 0.01). More importantly, compared to the control group, the PSQI score was lower in the study group after the intervention (*p* < 0.01, Figure 6).

### Treatment compliance between the two groups

Compared to the control group, treatment compliance was higher in the study group (*p* < 0.01, Table 2).

**Table 2:** Treatment compliance in both groups.

Groups	Cases	Complete compliance	Partial compliance	Non-compliance	Total compliance rate
Control group	60	24 (40.00)	25 (41.67)	11 (18.33)	49 (81.67)
Study group	60	28 (46.67)	30 (50.00)	2 (3.33)	58 (96.67)
χ^2^					6.98
*P*					<0.01

### Nursing satisfaction between the two groups

Compared to the control group, nursing satisfaction was higher in the study group (*p* = 0.01, Table 3).

**Table 3:** Nursing satisfaction in both groups.

Groups	Cases	Very satisfied	Basically satisfied	Dissatisfied	Total satisfaction rate
Control group	60	23 (38.33)	25 (41.67)	12 (20.00)	48 (80.00)
Study group	60	27 (45.00)	30 (50.00)	3 (5.00)	57 (95.00)
χ^2^					6.17
*P*					0.01

## Discussion

CHB is one of the most common infectious diseases in China (15). Despite the significant efforts made by the country and medical institutions, there is still a large number of CHB patients (16). Current medical treatments cannot completely cure hepatitis B; however, through nucleoside drugs and long-acting interferon therapy, patients can achieve relatively optimal control of HBV DNA, thereby significantly improving their survival time and quality of life (17). However, according to statistics, the self-care and psychological resilience abilities of Chinese patients with CHB are generally low, which, to some extent, affects treatment compliance, resulting in poor drug efficacy and recurrent disease progression (18). Therefore, effective intervention measures can improve the patient’s self-care abilities and help maintain a good psychological state, which is of significant benefit for disease control.

Phased focused nursing intervention is a clinical nursing model that respects individuals and explores their own potential and resources in the context of positive psychology (19). This intervention model focuses on the positive aspects of the individual, emphasizes their strengths and abilities, fully mobilizes the subjective initiative of patients, and stimulates their enthusiasm, thereby enhancing psychological resilience and improving self-care ability (20). Compared to the conventional intervention model, phased focused nursing is more humanized, targeted, and proactive (21).

In our study, the results showed that, compared to the control group, the levels of AST, ALT, and TBil were lower in the study group. These results suggest that phased focused nursing can improve liver function in CHB patients. By fully understanding the patient’s condition, phased focused nursing helps patients build confidence and face the disease with a positive and optimistic attitude and improves the overall treatment effect. Similarly, Yu et al. reported that targeted nursing interventions led to better recovery of liver function indicators in hepatitis B patients (22).

In our study, the results showed that, compared to the control group, the study group had higher CD-RISC, ESCA, and WHOQOL-BREF scores; higher scores for positive emotions and lower scores for negative emotions on the MUNSH; and lower PSQI scores after the intervention. These results suggest that phased focused nursing can improve psychological resilience, enhance sleep quality, promote self-care ability, increase subjective wellbeing, and improve quality of life in CHB patients. This is because by assessing patients’ self-care ability, identifying problems according to the content of the scale, and formulating reasonable intervention measures based on the assessment results, the approach addresses patients’ issues and improves their self-care ability. Moreover, phased focused nursing can comprehensively evaluate the actual condition of patients, understand their psychological state, and formulate targeted, phased, and individualized nursing measures to improve patients’ psychological resilience, enhance self-care ability, increase sleep quality, promote subjective wellbeing, and improve quality of life. Similarly, Lin et al. demonstrated that stage-based nursing for severe CHB patients enhanced self-care agency and reduced depression (23).

In addition, our study demonstrated that, compared to the control group, treatment compliance and nursing satisfaction were higher in the study group, suggesting that phased focused nursing can promote treatment compliance and nursing satisfaction in CHB patients, which is consistent with previous reports (24, 25).

## Conclusion

Phased focused nursing, with its structured and targeted approach, can effectively address knowledge gaps in comprehensive nursing for CHB patients. It not only significantly improves liver function but also has a profound impact on psychological resilience, sleep quality, self-care ability, subjective wellbeing, and quality of life. In addition, it enhances treatment compliance and increases nursing satisfaction, indicating its superiority over routine nursing in the multidimensional management of CHB patients.

## Data Availability

The raw data supporting the conclusions of this article will be made available by the authors, without undue reservation.
